# Rosai-Dorfman Disease Involving Multiple Organs: An Unusual Case with Poor Prognosis

**DOI:** 10.1155/2016/3920516

**Published:** 2016-10-31

**Authors:** Fandresena Arilala Sendrasoa, Irina Mamisoa Ranaivo, Onivola Raharolahy, Malalaniaina Andrianarison, Naina Harinjara Razanakoto, Lala Soavina Ramarozatovo, Fahafahantsoa Rapelanoro Rabenja

**Affiliations:** Department of Dermatology, Joseph Raseta Befelatanana Hospital, 101 Antananarivo, Madagascar

## Abstract

Rosai-Dorfman disease is a rare, benign histiocytic proliferative disorder that usually affects the lymph nodes. Although extranodal involvement has been reported in diverse sites, manifestation in the cardiovascular system is extremely rare. Specifically, cardiac involvement in Rosai-Dorfman disease is an extraordinarily infrequent event. We describe a case of a 36-year-old female who presented Rosai-Dorfman disease of multiple organs including the heart, with poor prognosis.

## 1. Introduction

Rosai-Dorfman disease (RDD), also known as “sinus histiocytosis with massive lymphadenopathy,” is a nonmalignant disease of histiocyte proliferation. The classical clinical presentation is with painless cervical lymphadenopathy; however, extranodal involvement is frequent, occurring in approximately 40% of cases. Extranodal RDD may involve a wide variety of anatomic sites, either as isolated or in association with lymphadenopathy. The majorities of extranodal RDD are encountered in the skin, nasal cavity and paranasal sinuses, orbit, upper respiratory tract, and bone [1]. Cardiac involvement (cRDD) has previously been documented as occurring in <0.1% of cases [2]. We report one case of RDD that involves multiple organs: skin, lymph nodes, pleura, and heart.

## 2. Case Presentation

**Figure 1:**
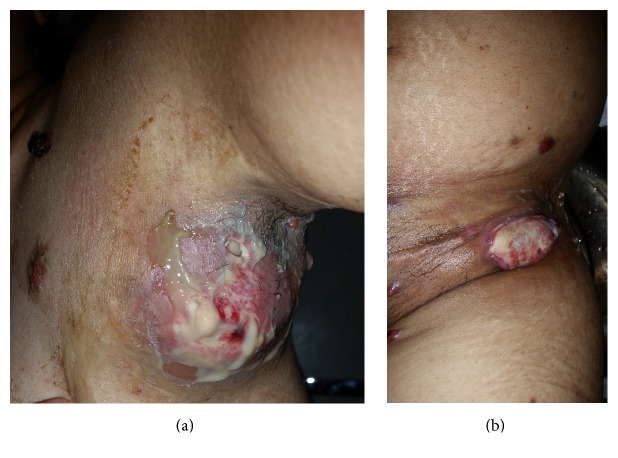
Large, ulcerative axillary, and inguinal lymphadenopathies.

**Figure 2:**
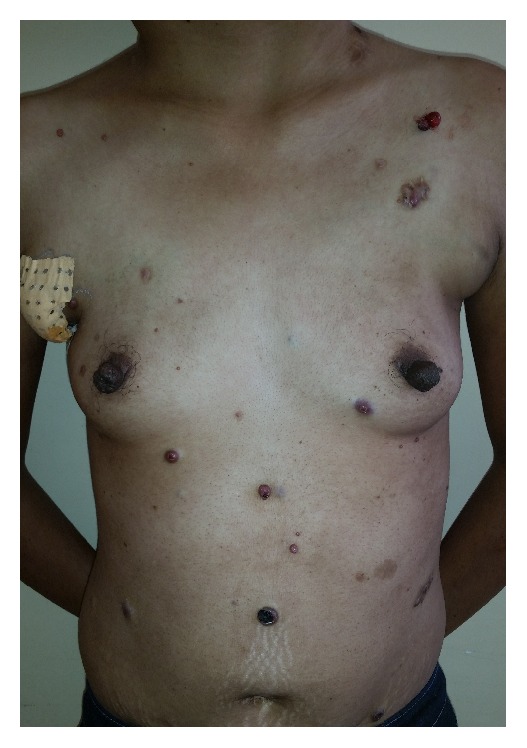
Disseminated multiples red brown papulonodules surrounded by satellite lesions on the trunk.

**Figure 3:**
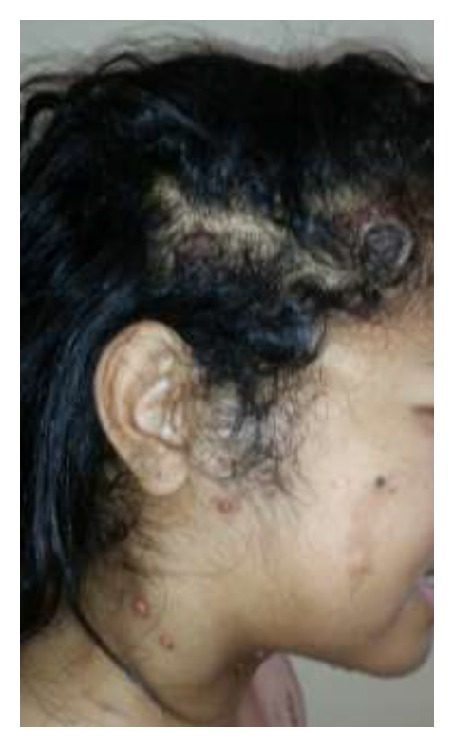
Ulcerated nodules on the scalp.

A 38-year-old woman, nonsmoker, without any significant past medical history presented with multiple lymph nodes which had progressed during the past year. She also had malaise, fever, anorexia, and weight loss. She had received antituberculosis treatment for these symptoms but no improvement was observed. No symptoms such as chest pain or dyspnea were complained by the patient. On physical examination, his temperature was 38°C with a pulse of 95 beats/min. She had bilateral painless cervical lymphadenopathies of different sizes (2–6 cm), which were nontender hard and fixed in deep tissue. Axillary and inguinal lymphadenopathies were large and ulcerative with pyogenic suppuration (Figures 1(a) and 1(b)). She presented with disseminated multiples red brown papulonodules surrounded by satellite lesions on the trunk (Figure 2) and ulcerated nodules on the scalp (Figure 3). The remainder of the examination, including the cardiovascular and respiratory systems and the abdomen, showed no significant findings. She had no hepatic or splenic enlargement on physical examination.

Her admission laboratory tests showed a white blood cell count of 22 350/mm^3^ (86% neutrophils and 10% lymphocytes) with a platelet count of 1 150 000/mm^3^, an ESR of 32 mm/h, and a C-reactive protein of 135 mg/L. Her lactate dehydrogenase was 230 IU/L (*n* = 100–250 IU/L). Serology for human immunodeficiency virus was negative.

**Figure 4:**
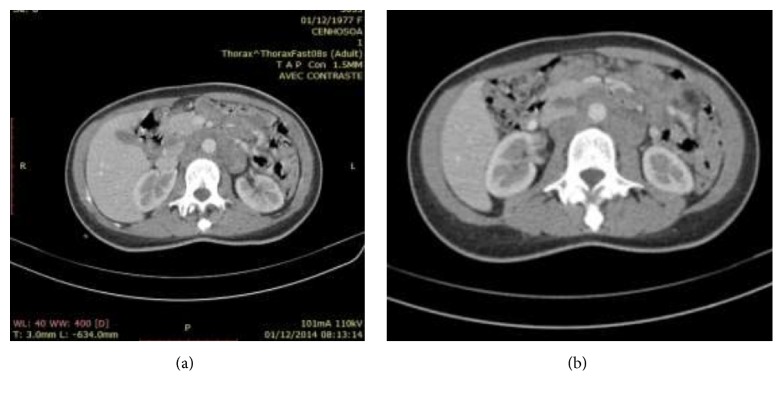
(a) Mesenteric lymph nodes. (b) Left pararenal lymph node.

Chest and abdominal CT scan revealed a moderate volume of left-side pleural effusion and mesenteric and pararenal lymph nodes (Figures 4(a) and 4(b)), respectively. Skin pathology showed emperipolesis. Lymph node pathology showed architectural disruption with polymorphic infiltrate composed of mainly foamy histiocytes and cytophagocytes; lymphoplasmocytes associated with neutrophils. Immunohistochemical stains showed S-100 and CD-68 positive histiocytes, but no staining for CD1a. The diagnosis of RDD was made. She received intravenous injection of methylprednisolone (2 mg/kg/d) for 3 days. On the third day of this treatment, she developed junctional tachycardia. Echocardiogram revealed septal hypertrophic with low LVEF 36% and noncompressive pericardial effusion, but no obvious nodules or masses were found. Because of circulatory collapse, electric cardioversion was required to restore sinus rhythm.

In spite of the low dose of corticosteroids, adequate management for cardiac involvement, and careful use of antibiotics, the patient died 2 months later from multiple organ dysfunction syndrome. Consent for autopsy was not obtained.

## 3. Discussion

Our patient's case illustrates an uncommon evolution of RDD disease. It is presumably the second reported case of RDD disease in Madagascar. The first case had an atypical clinical presentation but without visceral involvement, which responded well to corticosteroid treatment [3].

RDD is a disease whose status is largely relegated to an intriguing intellectual curiosity. Proposed mechanisms regarding the pathogenesis of RDD have been discussed, most relating to potential infectious agents like Herpesvirus 6 (HHV-6) and Epstein-Barr virus (EBV) and a disorder of immune regulation. RDD may occur in any age group, but most cases occur in the first or second decade [4]. There is a slight predilection for men (58%) and individuals of African descent [5].

RDD has been reported to involve many sites, including soft tissue sites, genitourinary tract, upper respiratory tract, skin, skeleton [6], gastrointestinal tract [7, 8], thyroid, head and neck region, orbit, and central nervous system [9]. The skin is one of the most common sites of extranodal RDD. Our case presented the typical cutaneous lesion in RDD. However, she presented with generalized lymphadenopathy which is a poor prognosis factor in RDD.

Extranodal manifestation of RDD in the heart is very rare. To date, eighteen individual cases have been described in the literature: 3 pediatric cases (<16 years of age) and 15 adult cases (≥16 years of age) [10]. Taking the current case into account, the overall median and average ages of adult patients with cardiac RDD at diagnosis were 53 years and 48.8 years (range, 12–79 years). There was a male predominance, with male to female ratio of 2 : 1. Fourteen cases had pure extranodal RDD, whereas five cases were in association with nodal RDD [8, 10, 11]. Three patterns were identified for the primary reported site of cardiac involvement: an intracardiac mass with or without underlying infiltration, pericardial/epicardial involvement, and a pulmonary arterial mass [10]. Our case presented mild pericardial effusion with infiltration. Her involvement of heart in RDD was difficult to confirm: heart biopsy was not made because there are no obvious nodules or mass and consent for autopsy was not obtained. However, other etiologies of tachycardia junctional were ruled out: ischemia, metabolism, and dysthyroidism.

Definitive diagnosis of RDD is based on histological assessment, whose cornerstones are identification of emperipolesis and appropriate immunohistochemical analysis. Emperipolesis related to RDD refers to the presence of histiocytes containing intact lymphocytes within their cytoplasm. Immunohistochemical staining for S100 protein is considered diagnostic; cells will also be positive for CD68 and negative for CD1a, helping to distinguish RDD from Langerhans cells [12].

At present, RDD is considered a benign or reactive proliferation showing spontaneous regression. Treatment does not seem necessary in the majority of patients because the disease does not usually threaten life or organ function. There is no ideal therapeutic regimen for this disease [13]. The treatment options range from surgery, radiotherapy, and corticosteroids to chemotherapy. Some patients with cardiac involvement of RDD have shown a good response to corticosteroid treatment. O'Gallagher et al. [10] reported a patient with intracardiac mass of RDD who was treated with corticosteroids and showed little change in the size of the mass. Chaitanya and Gupta [14] reported also a patient with RDD in whom cardiac involvement remitted with low dose corticosteroid plus surgery. Our patient did not show response to corticosteroid therapy; our case shows the severity of RDD. In rare cases, RDD can be locally aggressive and involve the vital organs, causing death [10]. Furthermore, death can also occur in patients with RDD who have generalized lymphadenopathy and immune dysfunction [6, 15].

## 4. Conclusion

Our case is unusual in that 4 organs or sites were involved, especially the heart. The nonresponse to corticosteroid therapy in this patient and her death showed that some subjects with RDD may have poor prognosis.
